# Sport in Town: The Smart Healthy ENV Project, a Pilot Study of Physical Activity with Multiparametric Monitoring

**DOI:** 10.3390/ijerph18052432

**Published:** 2021-03-02

**Authors:** Marco Laurino, Tommaso Lomonaco, Francesca Giuseppa Bellagambi, Silvia Ghimenti, Alessandro Messeri, Marco Morabito, Elena Marrucci, Lorenza Pratali, Maria Giovanna Trivella

**Affiliations:** 1 Institute of Clinical Physiology, National Research Council, 56124 Pisa, Italy; e.marrucci1@studenti.unipi.it (E.M.); lorenza@ifc.cnr.it (L.P.); trivella@ifc.cnr.it (M.G.T.); 2 Department of Chemistry and Industrial Chemistry, University of Pisa, 56124 Pisa, Italy; tommaso.lomonaco@unipi.it (T.L.); silvia.ghimenti@unipi.it (S.G.); 3 Institute of Analytical Sciences, University of Claude Bernard Lyon 1, Villeurbanne, 69100 Lyon, France; francesca.bellagambi@univ-lyon1.fr; 4 Institute of Bioeconomy, National Research Council (IBE-CNR), 50019 Florence, Italy; alessandro.messeri@unifi.it (A.M.); marco.morabito@ibe.cnr.it (M.M.); 5 Centre of Bioclimatology, University of Florence (UNIFI), 50144 Florence, Italy

**Keywords:** sport, multiparametric monitoring, air pollution, heat stress, microclimate determinants, heart rate, breath, physical activity

## Abstract

Background: Increasing evidence links meteorological characteristics and air pollution to physiological responses during sports activities in urban areas with different traffic levels. Objective: The main objective of the Smart Healthy ENV (SHE, “Smart Monitoring Integrated System For A Healthy Urban Environment In Smart Cities”) project was to identify the specific responses of a group of volunteers during physical activity, by monitoring their heart rates and collecting breath samples, combined with data on meteorological determinants and pollution substances obtained through fixed sensor nodes placed along city routes and remotely connected to a dedicated data acquisition server. Methods: Monitoring stations were placed along two urban routes in Pisa, each two km long, with one located within the park beside the Arno river (green route) and the other in a crowded traffic zone (red route). Our sample participants were engaged in sports activities (*N* = 15, with different levels of ability) and were monitored through wearable sensors. They were first asked to walk back and forth (4 km) and then to run the same route. The experimental sessions were conducted over one day per route. A breath sample was also collected before each test. A questionnaire concerning temperature and fatigue perception was administered for all of the steps of the study over the two days. Results: The heart rates of the participants were monitored in the baseline condition, during walking, and while running, and were correlated with meteorological and pollutant data and with breath composition. Changes in the heart rates and breath composition were detected during the experimental sessions. These variations were related to the physical activity and to the meteorological conditions and air pollution levels. Conclusions: The SHE project can be considered a proof-of-concept study aimed at monitoring physiological and environmental variables during physical activity in urban areas, and can be used in future studies to provide useful information to those involved in sports and the broader community.

## 1. Introduction

As stated by the European Environment Agency, “A clean environment is essential for human health and well-being. At the same time, the local environment can also be a source of stressors—for example air pollution, noise, hazardous chemicals—that negatively affect health.” The interactions between the environment and human health are highly complex and difficult to assess. Numerous environmental variables (such as ambient air pollution, poor water quality, and insufficient sanitation) are expected to affect outdoor physical activity (OPA) [1]. Chronic diseases are also known to be closely linked to unhealthy lifestyles. OPA can have direct health benefits, improving mental and physical wellbeing and health-related quality of life, and long-term adherence to physical activity is healthier for individuals [2,3,4]. Physical activity can be conducted in both city streets and urban parks and trails, which we refer to as “urban red areas” (URAs) and “urban green areas” (UGAs), respectively. UGAs enable a wide range of free or low-cost activities (both training and recreational), based on environmental factors that are frequently linked to increased levels of OPA [5,6]. In URAs, ambient fine particulate matter (PM_2.5_) and household air pollution from solid fuel combustion have been identified as risk factors that have negative effects on human health, similar to those reported for other habits such as smoking and drinking alcohol [7,8]. The effects of increased levels of air pollution on athletic performance have been measured using physiological parameters. In 2001, Carlisle and Sharp examined the effects of major pollutants on human health in terms of exercise [9]. They suggested that physical activity should not be conducted during rush hour, in order to minimize exposure to high levels of nitrogen oxides (NO_x_), carbon monoxide (CO), and volatile organic compounds (VOCs). These pollutants are likely to accumulate in the environment and thus may affect athletic performance. In addition, OPA is not recommended if ozone (O_3_) levels are high, as this results in a significant decrease in lung function [10,11]. Ambient levels of sulfur dioxide (SO_2_) can also be a significant irritant for asthmatics [11].

The cardiovascular benefits of exercise are well established. Exercising regularly reduces the risk of adverse cardiovascular events and can act as a multifunctional intervention tool for prevention, due to its effects on multiple biochemical pathways, unlike conventional drug therapy [12]. In particular, exercise training is an important additional type of non-pharmacological treatment for patients suffering from heart failure (HF), and has proven positive effects on mortality, morbidity, exercise capacity, and quality of life [13]. This implies that the OPA conducted in urban areas by amateurs, athletes, or those suffering from respiratory and heart diseases (such as HF), bears a risk of adverse effects from environmental pollution. Thus, the environmental monitoring of pollution in urban areas, both in terms of environmental variables (i.e., humidity and temperature) and pollutants (i.e., chemical air pollutants), should be conducted [14,15,16,17]. A multiparametric approach can provide information about both pollution levels and physical activity, thus helping those conducting OPA to select the most suitable days and the best urban areas, along with personalizing their training programs. Physical activity monitoring can be conducted through exhaled breath analysis, as the chemical composition of human breath samples enables relevant information about ongoing physiological processes to be obtained non-invasively [18,19,20]. During a typical respiratory cycle, several endogenous and exogenous VOCs are generally exchanged within the upper and lower respiratory tract, meaning that breath analysis is an effective approach to monitoring human exposure to environmental pollutants and health status [21]. The main advantage of this approach is the use of non-invasive collection procedures, as unlike blood collection they do not require trained personnel [22]. Breath acetone and isoprene are potentially useful indicators of the β-oxidation of fatty acids and of cardiac output, respectively [23].

This paper presents a proof-of-concept study involving the multi-parametric monitoring of healthy volunteers conducting OPA in urban areas, together with remotely acquired environmental variables. This can be regarded as the first step in a future plan for smart cities [24], and represents a starting point for examining the links between human health, physical activities, and environmental pollution, which can then be investigated in further detail with a larger sample.

Environmental conditions can have specific effects on different people, so the aim of this study is to lay the foundation for a personalized multi-parametric monitoring approach and the design of personalized training programs, by examining the responses of individuals with different health statuses and fitness levels. Thus, the main objectives of this study are to:

- Build a dedicated system to monitor environmental conditions (meteorological and pollution conditions) in different urban areas and individual physiological parameters during outdoor physical activity;
- Develop a pilot feasibility study for evaluating the impact of pollution and microclimatic variables on the populace.

## 2. Materials and Methods

**Figure 1:**
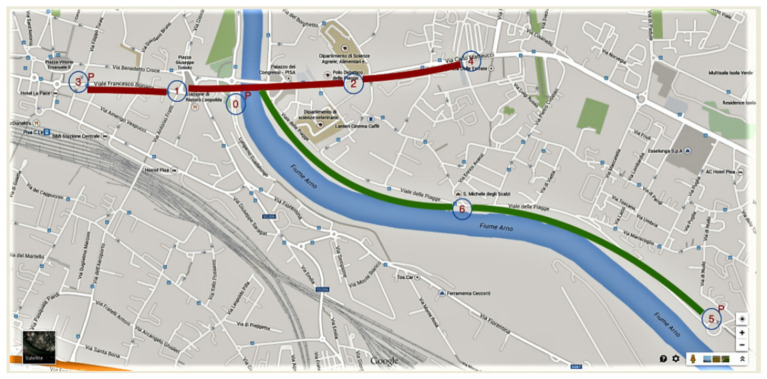
Fixed sensor nodes along the Arno River green park (green route) and along a crowded traffic zone (red route). Five nodes were positioned on the red route (nodes 0, 1, 2, 3, and 4). Node 0 was also on the green route, where two additional nodes were positioned (nodes 5–6).

Two routes were identified (A and B) in the city of Pisa. A “green route” was selected along the Arno river in an area characterized by extensive vegetation and limited vehicular traffic, and a busier “red route” was located along a high traffic-volume road artery (see Figure 1). Each route was approximately 4 km (round trip).

Fixed sensor nodes were placed in both these “urban environment” sectors to acquire meteorological and air pollution parameters and to collect air quality and thermal stress indicators.

Each fixed node can detect the concentrations of atmospheric pollutants typical of urban environments, such as CO, carbon dioxide (C0_2_), O_3_, and unburned hydrocarbons (HC), in addition to micro-meteorological parameters (i.e., air temperature, relative humidity, atmospheric pressure, wind speed, and black globe temperature). Some nodes also have a module for the detection of fine dust (PM_2.5_). The micro-meteorological parameters collected by the nodes enable the calculation of the wet-bulb globe temperature (WBGT) [25,26] and the universal thermal climate index (UTCI) [27,28], which can be used to evaluate the thermal environment and in particular the potential thermal discomfort to which the subjects were exposed during the tests.

The environmental and meteorological data were temporally matched with the physiological variables (heart rate changes) obtained by wearable sensors (WINPack system, Medical Equipment marked CE0434 according to 93/42/EEC directive, Medical Device Class IIA) and the chemical composition of the breath samples was collected before and after walking and running, for both the A and B paths. The wearable sensors of the WINPack enable the monitoring of multiple physiological parameters through different modules and include a rechargeable battery and a Bluetooth communication system, four leads, an electrocardiogram cable, a body position monitor, and a three-axial accelerometer module for measuring physical activity. The recorded data were post-processed and analyzed on a dedicated console by medical doctors, with the temporal matching of the environmental data acquired using a dedicated server (see Supplementary File S1 “ICT infrastructure”).

A questionnaire concerning temperature perception and fatigue was administered to the participants for all of the steps of the study (before the test, after walking, and after running) on both days. This was used to capture their subjective evaluations of physical fatigue, respiratory distress (difficulty in breathing), tachycardia, and possible thermal discomfort (see the Supplementary File S2 “Questionnaire”).

The protocol was approved by the North West Area Ethics Committee of the Tuscany Region (CEAVNO, Autonomous Section of the Regional Ethics Committee for Clinical Trials, Resolution AOUP 838/2013).

A small group of healthy volunteers was enrolled (*N* = 15). Each participant gave their informed consent. The data of the subjects enrolled were added to a specifically constructed database, which was managed exclusively by the medical researchers participating in the project.

The wearable sensors were positioned on the volunteers in an ambulatory room. After uploading the demographic data and checking the electrocardiogram (EKG) trace on the console, the volunteers (usually two) were accompanied to the chosen urban area on the first day (the same procedure was repeated on the second day in the other zone) to embark on the walking and running activities.

**Figure 2:**
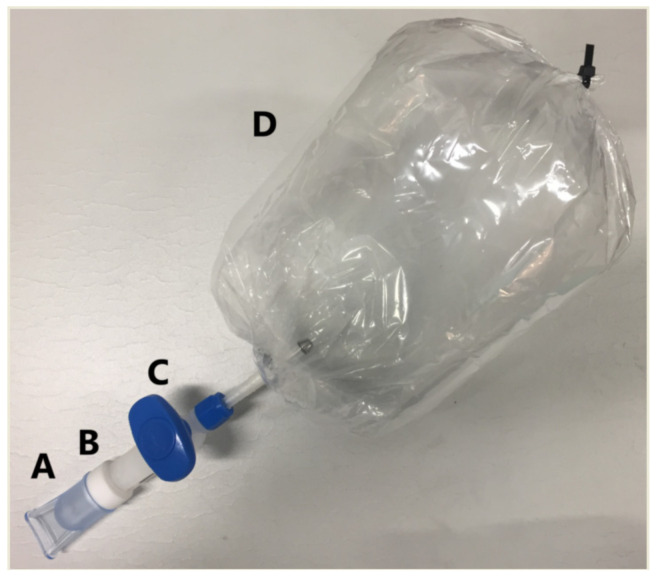
Breath sampling system composed of (**A**) disposable mouthpiece, (**B**) non-return valve, (**C**) two-way valve, and (**D**) Nalophan bag.

At the test site, the volunteers were asked to fill a custom-made Nalophan bag (Figure 2) so we could collect an exhaled breath sample. The Nalophan bag (50 cm × 23.5 cm, surface-to-volume ratio of 0.6 cm^−1^) was made from a roll of polyethylene terephthalate tube (diameter 23.5 cm, film thickness 20 μm) supplied by Kalle (Germany) according to the procedure described elsewhere [29].

A solid-phase extraction (SPE) technique was used to extract analytes from the breath samples. The analysis was conducted using a thermal desorption unit combined with a gas-chromatography and mass spectrometry (TD-GC-MS) procedure, as described elsewhere [30]. At the end of the test, the wearable sensors were removed so the acquired signal could be downloaded to the console for subsequent storage and analysis.

The correlations between the heart rate data (considering the RR interval, i.e., the time elapsed between two successive R-waves of the EKG signal) and the climate/pollutant data were statistically evaluated using non-parametric tests (paired Spearman rank correlations). To evaluate the effects of the run/walk conditions and red/green routes, we obtained correlations by separately considering the data collected in the four conditions. The data collected through questionnaires concerning temperature perception and fatigue were analyzed using repeated-measures ANOVAs (rANOVAs). The questionnaire data collected after walking (t1) and running (t2) for both the red and green routes were considered as variables in the rANOVAs, normalized with data collected before walking and running (t0). A main time effect (2 levels: “t1-t0,” which is the normalized post-walk, and “t2-t0,” which is the normalized post-run) and a route effect (2 levels: red or green) were considered as within-factors in the rANOVA.

## 3. Results

The Smart Healthy ENV (SHE) pilot study was conducted on a group of 15 healthy volunteers (age: 31 to 57 years old, mean age = 43 years old; six females and nine males; BMI: 19.6 to 27.0 kg/m^2^; mean BMI = 22.7 kg/m^2^), before and during four tests: walking along the green route, running along the green route, walking along the red route, and running along the red route. The experimental sessions were performed over nine days in 2017, from May to July.

Table 1 reports the demographic data and heart rate values of the enrolled population collected before and during the tests for both the A and B routes. Detailed information regarding each subject’s profession, training practice, smoking habits, and temperature and fatigue perception before and after each test were collected (see Supplementary File S3: “Subject Features” and File S4 “Questionnaire Data and Analysis”).

**Figure 3:**
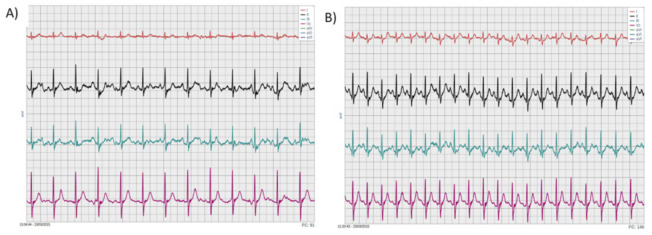
Typical examples of EKG monitored during walking (**A**) and running (**B**).

The instrumentation used in this pilot study enabled physiological data to be effectively collected during all of the tests. Figure 3 shows the electrocardiogram of a physically fit volunteer recorded during walking (panel A) and during running (panel B) along the green route.

The environmental sensor nodes were able to detect concentrations of CO, CO_2_, HC, O_3_, and PM_2.5_. The WBGT index and UTCI values were also estimated using the micro-meteorological parameters collected by each node.

The average values for each of the environmental and pollutant parameters were monitored and collected during the days of the field tests, and the parameters recorded for each subject when running and walking are shown in Table 2.

**Figure 4:**
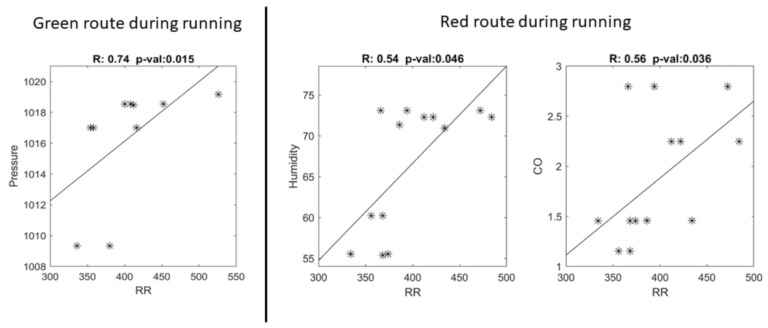
On the left is the scatterplot of the significant correlation between data during running for the green route (RR interval and atmospheric pressure), and on the right are the significant correlations between data during running for the red route (RR interval and relative humidity, and RR interval and CO concentration). The scatterplots report the lines of best fit, the Spearman’s correlation coefficients (R), and associated *p*-values (*p*-value). The RR interval is expressed in msec, atmospheric pressure in hPa, relative humidity in percentage, and CO in ppm.

Figure 4 shows the scatterplots of the significant correlations between heart rate and meteorological/pollutant data. A positive and significant correlation between the RR interval and atmospheric pressure was found (R = 0.74, *p*-value = 0.015) for running on the green route, whereas for running on red route, positive and significant correlations between the RR interval and relative humidity (R = 0.54, *p*-value = 0.046) and CO concentration (R = 0.56, *p*-value = 0.036) were found. No significant correlations between heart rate and meteorological/pollutant data were found during walking for either the red or green routes.

Exploratory correlations between the derived cardiac parameters (heart rate variability indices) and the meteorological and pollutant data are reported in the Supplementary File S5: “Exploratory Statistical Analysis.”

The pre-test normalized variables derived from the analysis of temperature perception and fatigue, such as “palpitations,” “sweating,” “general thermal sensation,” “local thermal sensation” (of the face, back, anterior chest, abdomen, arms, hands, legs, and feet), and “effort level” increased significantly from walking to running (*p*-value < 0.001 for all variables). The increases did not change with the red or green routes. “Chills” was the only variable that remained stable between walking and running. The Supplementary File S4, “Questionnaire Data and Analysis”, provides the complete rANOVA results.

The pollutant parameters generally revealed higher mean values of CO, CO_2_, and PM_2.5_ for the red route than for the green route, during both the walking and running tests. A similar pattern was observed for the mean HC value during the running tests, whereas during walking the mean HC value was slightly higher for the green than the red route. Higher O_3_ values were generally detected for the green rather than the red tests. The parameters related to air quality—CO, CO_2_, O_3_, and PM_2.5_—always remained below the reference thresholds indicated by the Regional Agency for Environmental Protection of Tuscany (ARPAT).

In terms of the microclimatic conditions, the days when the tests were conducted were characterized by clear or partly cloudy skies, with a solar radiation maximum of 756 watt/mq and a mean value of 623 watt/mq during the walk, and a 1072 watt/mq maximum and a mean value of 979 watt/mq during the run. The wind speed was weak during all test days, both for the walk and the run (below 2 m/s). The walking sessions were conducted early in the morning (from 8:00 to 10:00), and the air temperature ranged between 15.1 °C (3 May 2017) and 27.7 °C (7 July 2017), whereas the running test sessions were conducted later (from 10:00 to 12:00) when the air temperature values were higher, with peaks close to 30 °C on 7 July. The relative humidity was relatively high, with average values just over 60% during both the walking and running tests. Thus, the days selected to conduct the tests represented typical spring and summer microclimate conditions for the city of Pisa. These conditions often led to intense heat stress being observed in most of the tests, particularly during running. The UTCI confirmed average-to-moderate heat stress conditions during walking and strong heat stress conditions during running. Based on the average UTCI, 50% of the walking tests were carried out in strong heat stress conditions, about 27% in moderate heat stress conditions, and 23% with no thermal stress. About 77% of the running tests were carried out in strong heat stress conditions and the rest under moderate heat stress. The UTCI recorded mean values close to 32.5 °C during the walk and 36 °C during the run. The thermal stress evaluated by the WBGT index for about 50% of the running tests exceeded the threshold of 27.9 °C, with values nearing 28 °C during walking and 31 °C during running.

**Figure 5:**
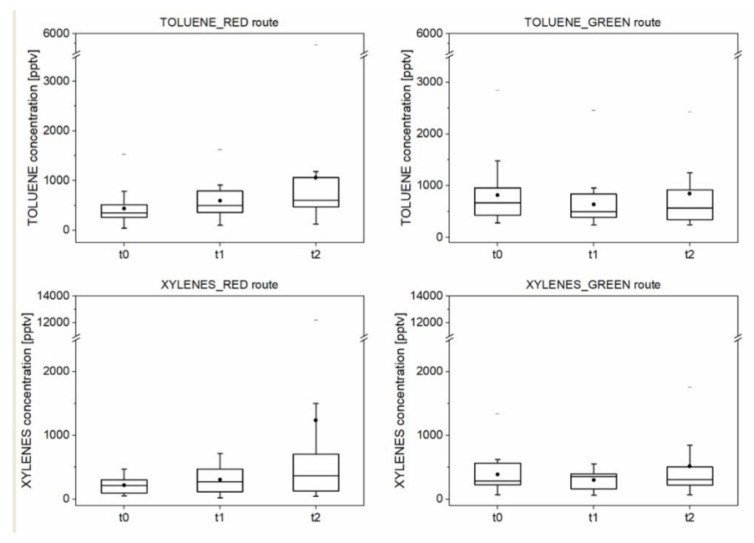
Box-plot of the concentration (expressed in pptv) of the traffic-related VOCs measured in the breath samples (*n* = 15 subjects) collected at the established times before the walk (t0), at its end (t1), and after the run (t2), during the tests on both green and red routes. Note: The box-plot shows the minimum, the 5th and the 25th percentiles, the median, the 75th and 95th percentiles, and the maximum values for each variable investigated. The dot inside the box shows the mean value.

**Figure 6:**
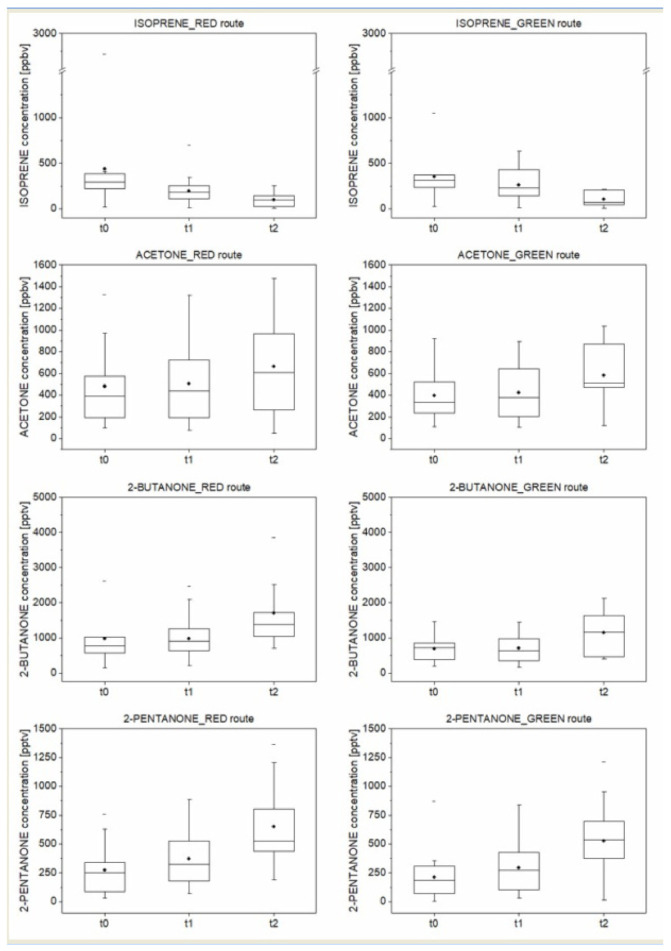
Box-plot of the concentration (expressed in pptv) of metabolic/oxidative stress-associated VOCs measured in the breath sample (*n* = 15 subjects) collected at the established times before the walk (t0), at its end (t1), and after the run (t2), during the tests on both green and red routes. Note: The box-plot shows the minimum, the 5th, and the 25th percentiles, the median, the 75th, and 95th percentiles, and the maximum values for each variable investigated. The dot inside the box shows the mean value.

Figure 5 and Figure 6 show the concentrations of traffic-related pollutants (i.e., toluene and total xylenes (meta-, orto-, and para-xylene)) and metabolic and oxidative stress-associated VOCs (i.e., isoprene, acetone, 2-butanone, and 2-pentanone) detected and measured within the breath samples.

## 4. Discussion

The environmental sensor nodes were able to detect the concentrations of carbon monoxide (CO), carbon dioxide (CO_2_), unburned hydrocarbons (HC), ozone (O_3_), and fine particulate matter (PM_2.5_), which were temporally matched with the activities of each volunteer. Higher concentrations of most of the pollutants monitored in the study were observed for the red route, which supports the assumption that urban parks and urban green areas in general can reduce concentrations of most pollutants, but also reveal increased O_3_ levels [31], which were also found in the monitored data, which revealed higher O_3_ values for the green rather than for the red route. Kuttler and Strassburger revealed an increase in O_3_ concentration in urban green areas in comparison to the nearby built up area during the summer [32]. Other studies conducted in Italy and in the Tuscany region have shown higher surface levels of O_3_ under the canopies of certain tree species [33] and a high potential for ozone formation in the main park of the city of Florence, which is linked to the presence of specific plants [34]. These elevated O_3_ levels result from the photochemical reaction of VOCs such as isoprene and monoterpenes, which can contribute to O_3_ formation under high solar radiation, elevated air temperature values, and weak wind speeds [35,36]. This phenomenon is more pronounced during the summer due to high solar radiation.

The microclimate conditions observed during the tests, and in particular those characterized by a WBGT threshold often exceeding 27.9 °C during the running tests, represents the limit beyond which an unacclimated subject must take care when performing competitive and long-duration sports activities, according to [37]. This WBGT-related heat stress risk threshold was also applied in a recent study [38] that aimed to estimate the impact of climate change, and therefore of high temperatures, on the potential performance of athletes during the Olympic Games in Tokyo that had been originally planned for the summer of 2020.

In this study, correlations between heart rate and the meteorological/pollutant data were only found during running conditions. A positive significant correlation between the RR interval and atmospheric pressure was found during the run on the green route. The reason for this relationship is not clear and it may have arisen by chance. Positive and significant correlations between the RR interval and relative humidity and CO concentration were found during the run on the red route. The increase in heart rates with relative humidity and CO concentration could be caused by the greater physical effort required due to environmental and pollution conditions, as they can affect physical activity. Interestingly, these correlations were not found for the green route, probably because of the effects of relative humidity and CO concentration on heart rate modulation. The levels of relative humidity and CO concentration for the green route sessions were lower on average than those of the red route sessions (see Table 2). Figure 4 also suggests a threshold mechanism for heart rate modulation with relative humidity and CO concentration.

From the analysis of temperature perception and fatigue recorded in the questionnaires, higher incidences of “palpitations,” “sweating,” “general thermal sensation,” “local thermal sensation” (for the face, back, anterior chest, abdomen, arms, hands, legs, and feet), and “effort level” were reported in the post-run condition than in the post-walk condition. The specific routes did not appear to affect the perception of temperature and fatigue. Although the results are not significant, it is interesting to note that the post-run condition in the red route demonstrated higher values than in the green route for “palpitations,” “sweating,” “local thermal sensation” (for the face, back, abdomen, and legs), and “effort level.” In general, these differences could be attributed to greater discomfort after the run for the red route than for the green route.

However, the results must all be interpreted with caution, because of the small sample size in the study and the large number of variables collected.

Thus, in this proof-of-concept study, breath analysis was used to evaluate the proposed personalized monitoring of exposure to environmental pollutants and of physical performance, through monitoring the variations of traffic-related pollutants (i.e., toluene and total xylenes (meta-, orto-, and para-xylene)) and other metabolic and oxidative stress-associated VOCs (i.e., isoprene, acetone, 2-butanone, and 2-pentanone) induced by performing physical activity along the red and green routes.

The results highlight that immediately after the walks and the runs in particular, the breath-exhaled toluene and total xylene concentrations were slightly higher (*p*-value < 0.05) than the pre-exercise levels for the red route (Figure 5), whereas breath concentrations of these compounds were not markedly different (*p*-value > 0.05) at each collection time for the green route (Figure 5). The increasing trend in the concentration of the traffic-related VOCs when exercising in polluted environments was not surprising, as the increase in minute ventilation during exercise increases proportionally to the quantity of inhaled air pollutants [9]. The breath-exhaled VOC levels were found to be highly variable from subject to subject, and breath concentrations after exercising on high-traffic routes were not statistically significantly higher than the low-traffic route levels. In fact, when breath composition was monitored for each volunteer, the concentration of traffic-related pollutants showed a more pronounced variation, confirming the usefulness of personalized monitoring, as reported elsewhere [39,40].

Regardless of the route, increases in breath concentrations for the oxidative stress-associated VOCs (i.e., acetone, 2-butanone, and 2-pentanone) from the pre-exercise state were observed, whereas the isoprene breath concentrations showed a decreasing trend as the intensity of physical activity increased (from rest to the walk up to the run) (Figure 6). The increase of acetone observed after exercise could be due to the rapid catabolism occurring during exercise [41]. Senthilmohan et al. observed a slight increase in breath acetone values with physical exercise, reaching up to 100–1400 ppb [42]. Similar results from King et al. confirm the presence of fat catabolisms during exercise [23].

The significant decrease (i.e., by at least a factor of three) in breath isoprene levels after exercise may be due to the increase in both the respiratory rate and cardiac output. The increase in respiratory rate leads to a decrease in the tidal volume with a concomitant increase in the ventilation of the upper airway area [43,44]. This scenario affects compounds like isoprene that are mainly present in alveolar air. The occurrence of oxidative stress may explain the increased production of 2-butanone and 2-pentanone [19] and thus their increased breath levels.

## 5. Conclusions

In conclusion, as the present study was a pilot evaluation of the feasibility of multiparametric monitoring, further extensive studies are required to define and conduct other assessments by considering different days in different seasons of the year.

## Figures and Tables

**Table 1:** Demographic data and heart rate values measured before walking (HR_b_) and before running (HR_pr_) for both green and red routes. Note: HR^max^_w_ and HR^max^_r_ are the maximum values reached during walking and running, respectively.

ID	Gender	Age (Year)	BMI (kg/m^2^)	Green Route				Red Route			
				HR_b_	HR^max^_w_	HR_pr_	HR^max^_r_	HR_b_	HR^max^_w_	HR_pr_	HR^max^_r_
N01	M	46	22.4	50	67	51	150	56	60	50	120
N02	M	39	22.2	65	70	68	162	64	67	65	150
N03	M	52	21.5	50	77	63	127	60	90	64	142
N04	M	44	21.5	63	82	67	155	60	90	65	127
N05	F	39	20.5	63	72	60	180	64	70	65	170
N06	F	49	20.7	74	90	75	120	77	105	80	170
N07	M	32	25.1	80	90	75	195	71	88	70	195
N08	M	32	27.0	50	78	71	140	54	75	61	170
N09	M	32	26.1	107	110	105	127	105	108	103	120
N10	F	57	19.6	64	100	80	172	85	115	94	180
N11	F	50	21.6	67	90	66	177	67	90	67	165
N12	F	37	23.5	80	105	84	172	86	112	80	180
N13	F	52	21.6	85	97	90	168	80	97	78	180
N14	M	31	24.7	75	85	75	172	77	82	52	160
N15	M	52	22.3	67	72	63	150	65	70	68	152

**Table 2:** Mean values of environmental parameters collected by the nodes during the tests for each experimental session of green and red routes. Route: R = red, G = green; WS = wind speed (m/s); T = temperature (°C); RH = relative humidity (%); P = atmospheric pressure (hPa); CO = carbon monoxide (ppm); CO_2_ = carbon dioxide (ppm); HC = hydrocarbons (ppm); O_3_ = ozone (ppb); PM_2.5_ = particulate matter (ug/m^3^); Rmed = average solar radiation (W/m^2^); Rmax = maximum solar radiation (W/m^2^); WBGTmed = average wet bulb globe temperature (°C); WBGTmax = maximum wet bulb globe temperature (°C); UTCImed = average universal thermal climate index (°C); UTCImax = maximum universal thermal climate index (°C). MD: missing data.

Walk																
Route	Date	WS	T	RH	P	CO	CO_2_	HC	O_3_	PM_2.5_	R Med	R Max	WBGT Med	UTCI Med	WBGT Max	UTCI Max
**R**	3 May	1.1	15.1	76	1018	2.6	380	3.1	40.9	MD	450	585	16.1	23.5	17.0	26.0
**R**	5 May	1.9	16.3	82	1020	2.5	415	6.3	42.2	18.5	450	605	17.1	23.0	17.9	25.9
**G**	1 June	1.1	23.5	58	1019	1.7	363	4.0	49.7	14.5	542	756	22.8	31.7	24.2	34.6
**G**	6 June	1.3	22.9	67	1008	1.9	362	4.1	51.0	3.8	334	535	21.7	28.0	22.8	31.2
**R**	7 June	2.0	21.7	54	1012	1.8	400	3.9	27.2	4.6	510	689	19.8	27.5	20.7	30.3
**G**	21 June	0.8	26.9	59	1016	1.6	345	5.0	48.3	5.7	491	651	25.8	34.5	26.9	36.6
**R**	23 June	2.3	27.0	67	1018	1.4	419	4.0	37.9	10.0	453	638	25.5	32.6	26.3	35.1
**G**	7 July	0.7	27.7	46	1018	1.6	361	4.3	58.7	3.0	446	600	25.0	33.8	26.1	35.9
**R**	14 July	1.3	27.6	72	1009	1.2	314	4.4	41.3	12.3	434	595	27.0	35.0	27.9	37.1
**Run**																
**Route**	**Date**	**WS**	**T**	**RH**	**P**	**CO**	**CO**_**2**_	**HC**	**O_3_**	**PM_2.5_**	**Rmed**	**Rmax**	**WBGT Med**	**UTCI Med**	**WBGT Max**	**UTCI Max**
**R**	3 May	0.8	16	72	1019	2.2	418	3.8	38.7	MD	830	1072	20	30.3	21.7	33.8
**R**	5 May	1.9	17.4	73	1021	2.8	420	6.6	41.2	14.5	786	944	19.2	29.3	20.1	31.4
**G**	1 June	0.7	25.6	50	1018	1.9	371	5.1	54.8	8	875	974	26.7	37.6	27.4	38.7
**G**	6 June	1.5	23.2	66	1009	1.8	336	3.3	48.1	3	606	1009	23.2	32.1	25.3	37.1
**R**	7 June	1.9	21.9	55	1013	1.5	333	3.5	39.3	3	844	903	22	33	22.3	33.8
**G**	21 June	0.5	27.8	53	1017	1.5	322	4.5	55.2	1.8	856	904	29.5	39.6	29.9	40.1
**R**	23 June	1.1	27.8	60	1019	1.2	428	3.9	35.8	9	888	1035	28.8	40.2	29.7	41.9
**G**	7 July	0.8	29.7	39	1018	1.5	343	5.0	57.6	6	812	939	28	39.9	28.8	41.4
**R**	14 July	1.3	27.8	71	1009	1.5	343	5.3	35.2	13	836	1030	29.2	40.1	30.3	42.3

## Data Availability

The data presented in this study are available on request from the corresponding author.

## Supplementary Materials

The following are available online at https://www.mdpi.com/1660-4601/18/5/2432/s1, File S1: ICT infrastructure; File S2: Questionnaire; File S3: Subject Features; File S4 Questionnaire Data and Analysis; File S5: Exploratory Statistical Analysis
