# Supplementary material for: Sport in Town: The Smart Healthy ENV Project, a Pilot Study of Physical Activity with Multiparametric Monitoring
Source: Int J Environ Res Public Health. 2021 Mar 2;18(5):2432. doi: 10.3390/ijerph18052432 (PMC7967572; doi:10.3390/ijerph18052432)
Supplement: Supplementary file 1 [file ijerph-18-02432-s001.zip › ijerph-1100318-suppl.-final/File S1.pdf]

ICT infrastructure

One of the main objectives of the Smart HealthyENV [www.progettoshe.it] project consist of developing a low-cost, highly scalable, efficient and widely distributed wireless sensor network (WSN) complementary to traditional weather stations and to existing air pollution measurement systems [Bacco et al. 2017]. The sensor node prototype, adaptable and easily expandable, has high processing capacity, a Wireless MBus radio module (WMBus) [Hersent et al. 2011] for data exchange between nodes, solar panel for internal battery charging, analog and digital programmable components to interface with on-board sensors. The data collected by the sensors are sent to a remote server to be validated and saved in a database.
In this study, 7 fixed gas nodes have been used; 3 of the 7 nodes also have a module for the detection of fine dust (PM 2.5). The nodes were placed along the two routes in the center of Pisa: 5 on the red route (characterized by high vehicular traffic) and 2 on the green route (tree-lined and less busy) that intersects with the red route.

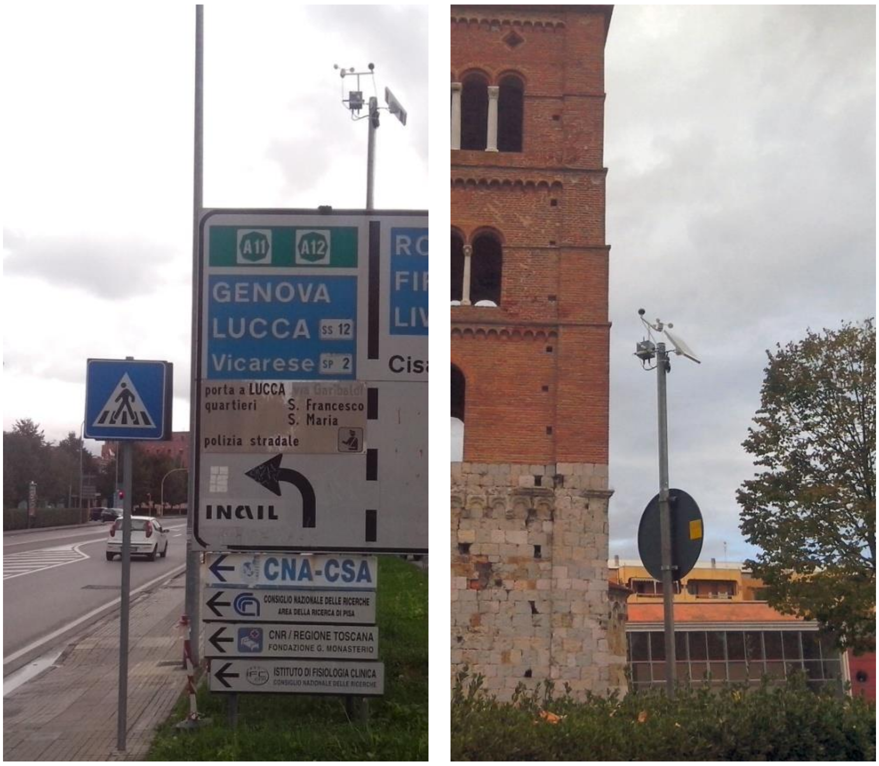

Gas nodes in red route (left) and green route (right)

The Wireless Sensor Network (WSN), developed by Infomobility Srl, consists of distinct sensor nodes that have a hardware board in common (S5P5, also made by Infomobility) that houses specific electronic components to implement various functionalities:
• Cortex-M3 32-bit CPU
• Power Management System
• Energy Harvesting system, with MPPT technology, for solar panel power supply.
• Local storage system (Storage): each board has an integrated 64KB EEPROM chip and an 8MB Flash memory. In addition, there is a microSD memory card connector that can hosts cards with capacity up to 4GB. The local storage allows to store the collected data by the sensors, until the trasmission to another node in the network, to a gateway node or outside the network.
• Communication interface. A modular approach was used for the radiofrequency communication system with the other network nodes. Various hardware modules, with compatible footprints, can be mounted; these modules shall support communication on frequencies ranging from VHF band (150 - 200 MHz) to the 5 GHz band. The modules typically host a second CPU integrating basic data exchange protocol (ZigBee , Bluetooth, WMBus).
• Analog and digital programmable interface that allows to use wide range of both analog and digital sensors, with direct interfacing or through an adaptation circuit.
The board, set up via firmware, makes the entire platform extremely flexible and adaptable to a large number of scenarios. The sensors used to detect pollutants and microclimatic parameters are:
• Sensirion SHT75 (temperature, relative humidity and dew point)

• Qbit-G3 NDIR gas sensor (CO, CO2, HC)
• SGX Sensortech MiCS-2614 (O3)
• Davis Intruments 7911 and WS1070 / WS (anemometers)
• Davis Intruments 7859 (rain gauge)
• Arcus RPTF 2 PT1000 (mean radiant temperature)
• Qbit MP25 (fine dust)
• Davis Instruments 6490 (radiometer)
• Freescale MPL3115A2 (atmospheric pressure)

Technical specifications of the commercial sensors are available online on the manufacturers' websites. The Qbit-G3 sensor was instead made by Qbit Optronics, according to Infomobility requirements, since, at the time of realization, no commercial sensors for the detecting CO, CO2, HC satisfied the requirements in term of minimum detectable limit and low cost. Electrochemical sensors were excluded due to their limited average lifetime, in favor of NDIR / DWR technology. The G3 sensor performs four IR measurements in four different optical bands; the response time is about tens of seconds (<1: 5 minutes). In order to obtain correct measurement conditions, it is necessary to allow the sensor to reach its operating temperature, obtained by feeding the sensor at least 10-15 minutes before starting a measurement cycle. The minimum detectable limit obtained under standard conditions are: <5ppm for CO, <10ppm for CO2 and <5ppm for HC, with a resolution of 1ppm for CO, 1ppm for CO2 and 1ppm for HC. All the sensors were treated in order to reduce the sensitivity to environmental disturbances demonstrated under measurement (temperature, humidity, pressure). The acquisition frequency of each node is 15 minutes. The collected data are sent to the coordinator node, which, in turn, transmits them to the server via GPRS connection. The nodes use the WMBus protocol (operating at 169 Mhz) for data exchange with the coordinator.
The types of nodes used during the tests of the work described:
• Fixed gas node: in this type of node the A / D programmable interface is used to connect: a) the NDIR module of the Qbit-G3 (for the detection of CO, CO2 and HC); b) the wind speed, temperature, relative humidity, dew point, average radiant temperature sensors; c) the acquisition board PTHO$_3$ (implemented for the prototype to connect the pressure, temperature, humidity and O3 sensors); d) for some nodes, the external module for detecting fine dust (PM 2.5). The node uses a WMBus protocol communication module.

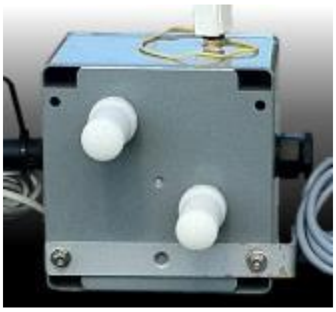

Gas sensor node Prototype

• Coordinator / gateway node: it has all the features and characteristics of the fixed gas node, but in addition, a GPRS/3G module for transmitting data to the server;

A proprietary application protocol has been implemented in order to allow data exchange between nodes and gateways and between gateways and servers, by transferring both messages concerning nodes status as well as frames of detected data by the sensors.

Software Platform

The software platform, hosted on a remote server, has the task of managing communication with the sensor network, making a first validation of the collected data and storing them in a database. Its main feature consists of being modulate and adaptable in terms of communication protocols with sensor networks as well as support different types and numbers of sensors, and in terms of collected data processing. All the data collected by the related sensors were extracted in CVS (Comma-Separated Values) format in order to provide the environmental conditions recorded by the sensor nodes. The software platform carries out a first check during data reception phase for outlier or out-of-range values, so that only reliable values of the micro environmental conditions are recorded and provided.
The software platform is based on the open source LAMP stack software (Linux, Apache, MySQL, PHP / Perl / Python) which is the main infrastructure for the development and use of highly scalable applications.

References

Bacco M, Delmastro F, Ferro E, Gotta A. 2017. Environmental monitoring for Smart Cities. IEEE Sens J 17(23):7767–7774, https://doi.org/10.1109/JSEN.2017.2722819.

O. Hersent, D. Boswarthick, and O. Elloumi, “M-Bus and Wireless MBus” The Internet of Things: Key Applications and Protocols, John Wiley and Sons 2011, pp. 153– 163
