# Supplementary material for: Sport in Town: The Smart Healthy ENV Project, a Pilot Study of Physical Activity with Multiparametric Monitoring
Source: Int J Environ Res Public Health. 2021 Mar 2;18(5):2432. doi: 10.3390/ijerph18052432 (PMC7967572; doi:10.3390/ijerph18052432)
Supplement: Supplementary file 1 [file ijerph-18-02432-s001.zip › ijerph-1100318-suppl.-final/File S2.pdf]

Perception questionnaire

ID ___________ Name: ____________ Surname: _______________

Date (day/month/year) : ____/…./……. Place: ____ ________

Time (Hour) (1-24): ____ minutes (0-60): ____

Activity: Running ☐ Walking ☐

1) Tell me your perception of:

Palpitations	not perceptible	mild	moderate	severe
Sweating	not perceptible	mild	moderate	severe
Chills	not perceptible	mild	moderate	severe

2) What level of general thermal sensation do you feel right now?
(Indicate with a X the chosen answer class)

1 ☐ **2** ☐ **3** ☐ **4** ☐ **5** ☐ **6** ☐ **7** ☐

Scale of the answer

From **1** COLD ⟶ to **7** HOT

Cold Discomfort 1 Intense 2 Moderate 3 Mild

No discomfort 4

Hot Discomfort 5 Mild 6 Moderate 7 Intense

3) What level of local thermal sensation are you feeling right now?

Face	1	2	3	4	5	6	7
Back	1	2	3	4	5	6	7
Anterior chest	1	2	3	4	5	6	7
Abdomen	1	2	3	4	5	6	7
Arms	1	2	3	4	5	6	7
Hands	1	2	3	4	5	6	7
Legs	1	2	3	4	5	6	7
Feet	1	2	3	4	5	6	7

3) What perception of effort level did you have during physical activity?
(Indicate with a X the chosen answer class)

**6**	Fatigue or null intensity	Similar to sitting or relaxing
**7**	Extremely light	A very easy movement
**8**		
**9**	Very light	Similar to a normal walk.
**10**		
**11**	Light	Comparable to the intensity of a light heating
**12**		
**13**	Hard enough	An intensity perceived as slightly challenging
**14**		
**15**	Hard	Intensity perceived as demanding
**16**		
**17**	Very hard	An intensity perceived as very demanding
**18**		
**19**	Extremely hard	A very high intensity that cannot be maintained
**20**	Maximum effort	A maximum intensity that is not very sustainable
