# Supplementary material for: Sport in Town: The Smart Healthy ENV Project, a Pilot Study of Physical Activity with Multiparametric Monitoring
Source: Int J Environ Res Public Health. 2021 Mar 2;18(5):2432. doi: 10.3390/ijerph18052432 (PMC7967572; doi:10.3390/ijerph18052432)
Supplement: Supplementary file 1 [file ijerph-18-02432-s001.zip › ijerph-1100318-suppl.-final/File S3.pdf]

ID	GENDER	PROFESSION	TRAINING SESSIONS			SMOKE HABIT	
			km/week	Hours/week	Days/week	Smokers	Ex-Smokers
N01	M	Researcher	70	7,00	4	0	0
N02	M	Researcher	20	7	3	0	0
N03	M	Osteopath	60-100	20	5	1	1
N04	M	Computer scientist	60	6	5	0	1
N05	F	Nurse	100	12	7	0	1
N06	F	Veterinary	40	6	3	1	0
N07	M	Researcher	10	3	2	0	0
N08	M	Researcher	72	6	6	0	0
N09	M	PhD student	2	3	3	0	0
N10	F	Researcher	15-20	3	2	1	0
N11	F	Teacher	20	3	3	1	0
N12	F	Employee	20-25	3	3	0	0
N13	F	Cardiologist	30	9	3-4	0	0
N14	M	PhD student	fitness		2	0	0
N15	M	Fireman	30	5	2-3	0	0
