# Supplementary material for: Sport in Town: The Smart Healthy ENV Project, a Pilot Study of Physical Activity with Multiparametric Monitoring
Source: Int J Environ Res Public Health. 2021 Mar 2;18(5):2432. doi: 10.3390/ijerph18052432 (PMC7967572; doi:10.3390/ijerph18052432)
Supplement: Supplementary file 1 [file ijerph-18-02432-s001.zip › ijerph-1100318-suppl.-final/File S4.pdf]

Questionnaire data

Before TEST (t0)

ID	ROUTE													
						Local thermal sensation								
		Palpitations (no=0)	Sweating	Chills	General thermal sensation	Face	Back	Anterior Chest	Abdomen	Arms	Hands	Legs	Feet	Effort level
N01	Red	0	0	1	2	4	4	3	3	2	2	3	3	6
N02	Red	0	0	2	2	4	4	4	4	2	2	3	3	6
N03	Red	0	0	0	3	4	4	4	4	3	3	3	4	6
N04	Red	0	0	0	4	4	4	4	4	4	4	4	4	6
N05	Red	0	0	0	4	4	4	4	4	4	4	4	4	6
N06	Red	0	0	0	3	3	4	3	4	4	4	3	4	6
N07	Red	0	0	0	4	4	4	4	4	4	4	4	4	6
N08	Red	0	0	0	4	4	4	4	4	4	4	4	4	6
N09	Red	0	1	0	4	4	4	4	4	4	4	4	4	6
N10	Red	0	0	0	4	4	4	4	4	4	4	4	5	6
N11	Red	0	1	0	5	5	4	5	4	4	5	4	5	6
N12	Red	0	0	0	4	4	4	4	4	4	4	4	4	6
N13	Red	0	0	0	5	5	5	5	5	4	5	4	5	6
N14	Red	0	0	0	4	4	4	4	4	4	4	4	4	7
N15	Red	0	0	0	5	5	5	5	5	4	4	4	4	6
N01	Green	0	0	0	4	4	4	4	4	4	4	4	4	6
N02	Green	0	0	0	3	4	4	4	4	4	4	3	4	7
N03	Green	0	0	0	4	4	4	4	4	4	4	4	4	6
N04	Green	0	0	0	3	3	3	3	3	3	3	3	3	6
N05	Green	0	0	0	4	4	4	4	4	4	4	4	4	6
N06	Green	0	0	0	3	3	3	3	3	3	3	3	3	6
N07	Green	0	0	0	4	4	4	4	4	4	4	4	4	6
N08	Green	0	0	0	4	4	4	4	4	4	4	4	4	6
N09	Green	0	0	0	4	4	4	4	4	4	4	4	4	6
N10	Green	0	0	0	4	4	4	4	4	4	4	4	5	6
N11	Green	0	0	0	4	4	4	4	4	4	4	4	5	6
N12	Green	0	0	0	4	4	4	4	4	4	4	4	4	6
N13	Green	0	0	0	4	4	4	4	4	4	4	4	4	6
N14	Green	0	0	0	4	4	4	4	4	4	4	4	4	6
N15	Green	0	0	0	4	4	4	4	4	4	4	4	4	6

Post – Walking (t1)

ID	ROUTE					Local thermal sensation								
		Palpitations (no=0)	Sweating	Chills	General thermal sensation	Face	Back	Anterior chest	Abdomen	Arms	Hands	Legs	Feet	Effort level
N01	Red	0	0	0	4	4	4	4	4	4	3	4	4	6
N02	Red	0	0	0	3	4	4	4	4	3	4	4	4	7
N03	Red	0	0	0	4	4	4	4	4	4	3	4	4	7
N04	Red	0	0	0	3	4	4	4	3	4	3	4	4	7
N05	Red	0	0	0	4	4	4	4	4	4	4	4	4	7
N06	Red	0	1	0	4	4	4	4	4	4	4	4	4	7
N07	Red	0	0	0	4	4	4	5	4	4	4	4	4	9
N08	Red	0	0	0	5	5	5	5	5	5	5	5	5	9
N09	Red	0	2	0	5	4	4	5	5	4	4	4	4	9
N10	Red	0	2	0	5	5	5	5	5	5	5	5	6	9
N11	Red	0	1	0	6	6	5	6	5	5	6	5	6	9
N12	Red	0	0	0	5	4	4	4	4	4	4	4	4	7
N13	Red	0	1	0	5	5	5	5	5	5	5	4	5	9
N14	Red	0	1	0	5	4	5	5	4	4	5	5	4	11
N15	Red	0	1	0	5	5	5	5	5	5	5	4	5	9
N01	Green	0	1	0	4	4	4	5	5	4	4	4	4	7
N02	Green	0	1	0	4	4	4	5	5	4	4	4	4	7
N03	Green	1	1	0	5	5	5	6	5	4	4	4	5	9
N04	Green	0	1	1	3	3	3	3	3	3	3	3	3	7
N05	Green	0	0	0	5	5	4	4	4	4	5	4	4	6
N06	Green	0	1	0	4	4	4	4	4	4	5	4	4	9
N07	Green	0	0	0	4	4	4	4	4	4	4	4	4	7
N08	Green	0	0	0	4	4	4	5	4	4	4	4	4	7
N09	Green	0	1	0	5	5	5	5	5	5	5	5	5	7
N10	Green	0	1	0	5	4	4	4	4	4	4	4	5	7
N11	Green	0	0	0	5	5	4	4	4	4	4	4	5	9
N12	Green	0	1	0	4	4	4	4	4	4	4	4	4	9
N13	Green	0	1	0	5	5	5	4	4	4	4	4	5	9
N14	Green	0	1	0	5	5	6	5	4	4	5	4	4	7
N15	Green	0	1	0	5	4	5	5	4	4	4	4	5	7

Post – Running (t2)

ID	ROUTE					Local thermal sensation								
		Palpitations(no=0)	Sweating	Chills	General thermal sensation	Face	Back	Anterior Chest	Abdomen	Arms	Hands	Legs	Feet	Effort level
N01	Red	0	1	0	5	5	5	5	5	4	4	4	4	8
N02	Red	0	2	0	5	6	6	6	6	4	5	4	5	13
N03	Red	1	2	0	5	5	5	5	5	5	4	4	5	11
N04	Red	1	2	0	5	5	5	5	5	5	5	5	5	12
N05	Red	0	0	0	4	4	4	4	4	4	4	4	4	6
N06	Red	1	2	0	6	6	6	6	6	6	6	6	6	12
N07	Red	0	2	0	5	5	5	6	5	5	5	5	5	13
N08	Red	0	2	0	6	5	6	6	6	6	6	6	6	11
N09	Red	1	2	0	6	6	6	6	6	6	6	6	6	12
N10	Red	0	3	0	6	6	6	6	6	6	6	6	6	14
N11	Red	2	2	0	7	7	6	7	7	6	7	6	7	15
N12	Red	2	2	0	6	6	6	6	6	6	6	6	6	12
N13	Red	1	2	0	6	6	6	6	6	6	6	6	6	12
N14	Red	1	2	0	6	6	6	6	5	5	5	6	5	13
N15	Red	2	2	0	6	6	6	6	6	6	5	5	6	15
N01	Green	0	2	0	6	6	6	7	7	7	5	5	4	13
N02	Green	0	2	0	6	6	6	5	6	6	5	6	5	11
N03	Green	2	3	0	6	6	5	5	5	5	5	5	5	13
N04	Green	2	3	0	5	6	5	5	5	5	5	5	5	13
N05	Green	1	2	0	5	6	5	5	5	5	5	5	5	11
N06	Green	0	3	0	5	6	5	5	5	5	5	5	6	13
N07	Green	1	3	0	5	5	5	5	6	5	5	5	5	13
N08	Green	0	2	0	6	6	6	6	6	6	6	6	6	13
N09	Green	2	2	0	6	7	6	6	6	5	5	6	5	11
N10	Green	1	1	0	6	6	5	5	5	5	5	5	6	15
N11	Green	2	2	0	6	6	5	5	5	5	5	5	6	13
N12	Green	2	2	0	6	7	6	6	6	6	6	6	6	13
N13	Green	2	2	0	5	5	5	5	5	5	5	5	6	12
N14	Green	1	2	1	6	6	6	5	5	6	5	6	5	13
N15	Green	0	2	0	6	6	6	6	5	5	4	6	6	11

Questionnaire data analysis

A repeated measures ANOVA was conducted considering as variables the questionnaire data after walk (t1) and run (t2), for both RED and GREEN route, normalized for the before walk and run conditions (t0).

Therefore, the ANOVA considered the normalized variables “t1-t0” and “t2-t0”. In the ANOVA, we considered a main time effect (2 levels: “t1-t0” that is the normalized post-walk and “t2-t0” that is the normalized post-run) and a route effect (2 levels: red or green) as within factor. In the table below the ANOVA results are reported.

*Table 1: The repeated measure ANOVA table. In the table are reported: the p-value of main effect of run/walk condition (**p-val (run/walk effect)**), the p-value of interaction effect of red/green rout (**p-val (red/green effect)**), the mean values and respective standard deviations of the normalized walk (**Walk (mean value, std)**) and run (**Run(mean value, std)**) conditions in both red and green routes, and the p-values of difference between walk and run conditions within red (**p-val (red walk vs red run)**) and green route (**p-val (green walk vs red run)**). ns stands for no significant (p-values>0.05)*

			RED			GREEN		
	p-val (run/walk effect)	p-val (red/green effect)	Walk (mean value, std)	Run (mean value, std)	p-val (red walk vs red run)	Walk (mean value, std)	Run (mean value, std)	p-val (green walk vs red run)
**Palpitations**	<0.001	ns	0.07 (0.05)	1.07 (0.21)	<0.001	0 (0.05)	0.8 (0.21)	<0.001
**Sweating**	<0.001	ns	0.73 (0.14)	2.2 (0.16)	<0.001	0.47 (0.14)	1.73 (0.16)	<0.001
**Chills**	ns	ns	0.07 (0.11)	0.07 (0.11)	ns	-0.2 (0.11)	-0.2 (0.11)	ns
**General thermal sensation**	<0.001	ns	0.67 (0.16)	1.87 (0.18)	<0.001	0.67 (0.16)	1.8 (0.18)	<0.001
**Face**	<0.001	ns	0.47 (0.13)	2.13 (0.18)	<0.001	0.27 (0.13)	1.47 (0.18)	<0.001
**Back**	<0.001	ns	0.47 (0.14)	1.6 (0.15)	<0.001	0.27 (0.14)	1.47 (0.15)	<0.001
**Anterior Chest**	<0.001	ns	0.6 (0.15)	1.53 (0.18)	<0.001	0.53 (0.15)	1.67 (0.18)	<0.001
**Abdomen**	<0.001	ns	0.33 (0.14)	1.6 (0.18)	<0.001	0.27 (0.14)	1.53 (0.18)	<0.001
**Arms**	<0.001	ns	0.13 (0.13)	1.53 (0.16)	<0.001	0.6 (0.13)	1.67 (0.16)	<0.001
**Hands**	<0.001	ns	0.33 (0.18)	1.2 (0.17)	<0.001	0.47 (0.18)	1.53 (0.17)	<0.001
**Legs**	<0.001	ns	0.2 (0.12)	1.6 (0.18)	<0.001	0.53 (0.12)	1.53 (0.18)	<0.001
**Feet**	<0.001	ns	0.33 (0.13)	1.4 (0.18)	<0.001	0.4 (0.13)	1.4 (0.18)	<0.001
**Effort level**	<0.001	ns	1.53 (0.3)	6.47 (0.49)	<0.001	2.07 (0.3)	5.87 (0.49)	<0.001
