# Supplementary material for: Sport in Town: The Smart Healthy ENV Project, a Pilot Study of Physical Activity with Multiparametric Monitoring
Source: Int J Environ Res Public Health. 2021 Mar 2;18(5):2432. doi: 10.3390/ijerph18052432 (PMC7967572; doi:10.3390/ijerph18052432)
Supplement: Supplementary file 1 [file ijerph-18-02432-s001.zip › ijerph-1100318-suppl.-final/File S5.pdf]

Statistical Analysis

In this addendum, the statistical results about the non-parametric Spearman's correlations are reported between cardiac features, during walking and running in green and red routes, and the meteorological/pollutant data. The statistical results are considered preliminary because of the low-sample size in our pilot study and the lack of multiple comparison correction.
From the collected EKG during walking and running in both routes, the following cardiac parameters about heart rate and heart rate variability are extracted:

- mean value of RR intervals (**RR**)
- standard deviation of RR intervals (**RRstd**)
- standard deviation of successive RR interval differences (**dRRstd**)
- root mean square of successive RR interval differences (**RMSSD**)
- mean number of successive difference of RR intervals differing by more of 50 msec divided by the total number of RR intervals (**pnn50**).
- ratio between the power spectrum densities (PSD) in low and high frequencies bands (**LF/HF**) of RR tachogram signal. The low frequency corresponds to the PSD in 0.04-0.15 Hz band and high frequency to the PSD in 0.15-0.4 Hz band.
- Shannon entropy of RR tachogram signal (**Shannon H**). It is a non-linear measures used to evaluate the distribution complexity of RR tachogram signal. Higher entropy describes a more irregular behavior of a signal.

The pollutant data considered for correlation with the cardiac parameters are:

- carbon monoxide (**CO**)
- carbon dioxide (**C02**)
- ozone (**O3**)
- unburned hydrocarbons (**HC**)
- fine dust of PM 2.5 (**pm25**)

The micro-meteorological data considered for correlation with the cardiac parameters are:

- air temperature (**Temp**)
- relative humidity (**Humidity**)
- atmospheric pressure (**Pressure**)
- wind speed (**Wind Speed**)
- radiant temperature (**Rtd**)

In the following figures, the scatterplots for each significant correlations between cardiac features and the meteorological/pollutant data are shown. For each correlation, the Spearman's correlation coefficient and associated p-value are reported.
Figure 1A shows the significant correlations for green route during walking, Figure 2A shows the significant correlations for green route during running, Figure 3A shows the significant correlations for red route during walking and Figure 4A shows the significant correlations for red route during running.
Considering the multi-parametric statistical analysis, we can not conclude any significant relationship between the extracted parameters for the limited number of subject in our pilot study and the lack of significant effect in the case of correction for multiple comparisons.

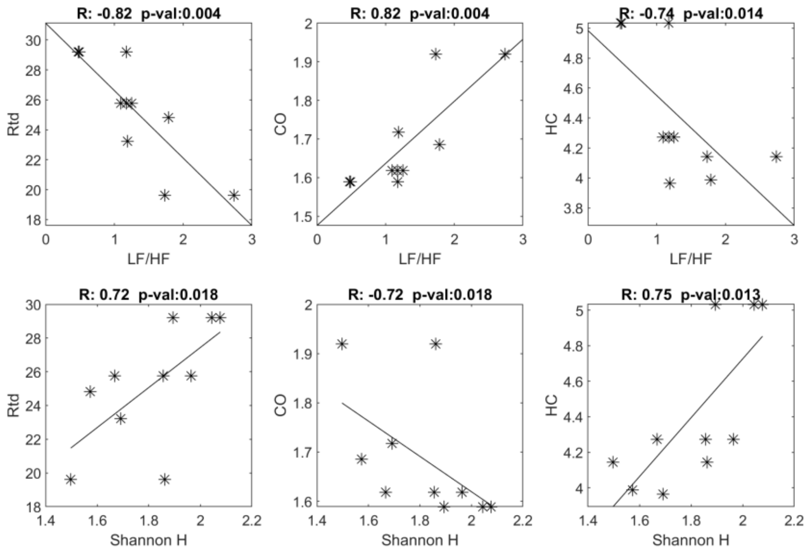

Figure A1 Significant correlations for green route during walking

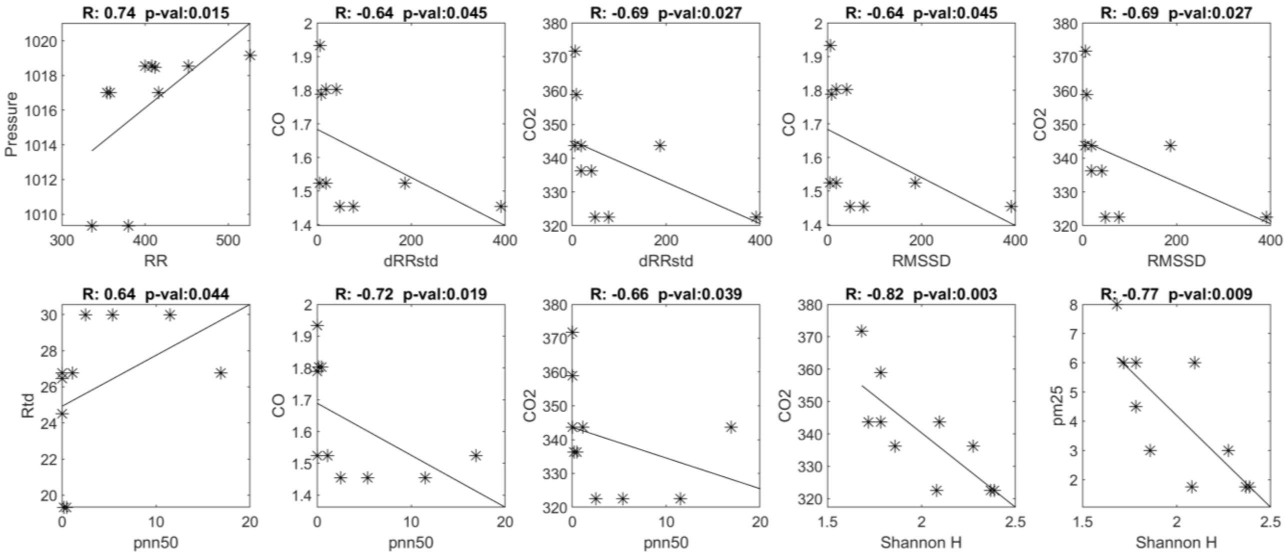

Figure A2 Significant correlations for green route during running

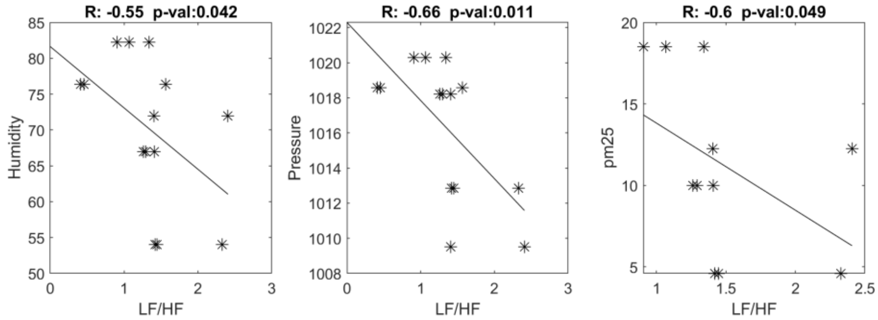

Figure A3 Significant correlations for red route during walking

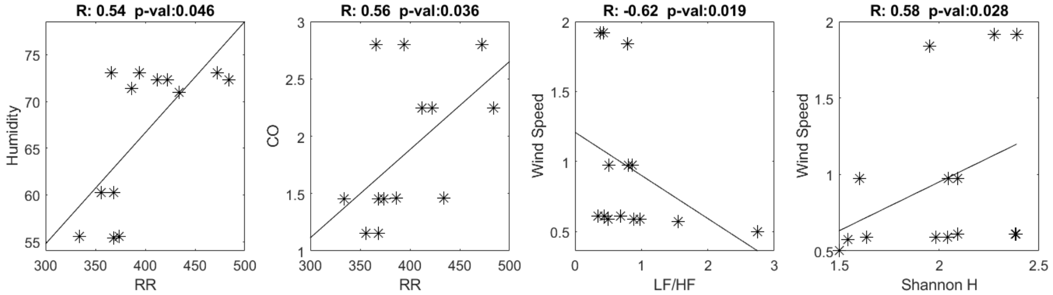

Figure A4 Significant correlations for red route during running
